# User Experience of Augmented Reality System for Astronaut's Manual Work Support

**DOI:** 10.3389/frobt.2018.00106

**Published:** 2018-09-12

**Authors:** Kaj Helin, Timo Kuula, Carlo Vizzi, Jaakko Karjalainen, Alla Vovk

**Affiliations:** ^1^VTT Technical Research Centre of Finland Ltd, Espoo, Finland; ^2^ALTEC SpA, Turin, Italy; ^3^Oxford Brookes University, Oxford, United Kingdom

**Keywords:** augmented reality, space domain, work support, user experience, Microsoft Hololens

## Abstract

This paper introduces Augmented Reality (AR) system to support an astronaut's manual work, it has been developed in two phases. The first phase was developed in Europeans Space Agency's (ESA) project called “EdcAR—Augmented Reality for Assembly, Integration, Testing and Verification, and Operations” and the second phase was developed and evaluated within the Horizon 2020 project “WEKIT—Wearable Experience for Knowledge Intensive Training.” The main aim is to create an AR based technological platform for high knowledge manual work support, in the aerospace industry with reasonable user experience. The AR system was designed for the Microsoft HoloLens mixed reality platform, and it was implemented based on a modular architecture. The purpose of the evaluation of the AR system is to prove that reasonable user experience of augmented reality can reduce performance errors while executing a procedure, increase memorability, and improve cost, and time efficiency of the training. The main purpose of the first phase evaluation was to observe and get feedback from the AR system, from user experience point-of-view for the future development. The use case was a filter change in International Space Station (ISS)—Columbus mock-up in the ESA's European Astronaut Centre (EAC). The test group of 14 subjects it included an experienced astronaut, EAC trainers, other EAC personnel, and a student group. The second phase the experiment consisted of an *in-situ* trial and evaluation process. The augmented reality system was tested at ALTEC facilities in Turin, Italy, where 39 participants were performing an actual real astronaut's procedure, the installation of Temporary Stowage Rack (TSR) on a physical mock-up of an ISS module. User experience evaluation was assessed using comprehensive questionnaires, and interviews, gathering an in-depth feedback on their experience with a platform. This focused on technology acceptance, system usability, smart glasses user satisfaction, user interaction satisfaction, and interviews, gathering an in-depth feedback on their experience with a platform. The analysis of the questionnaires and interviews showed that the scores obtained for user experience, usability, user satisfaction, and technology acceptance were near the desired average. Specifically, The System Usability Scale (SUS) score was 68 indicating that the system usability is already nearly acceptable in the augmented reality platform.

## Introduction

This paper introduces AR system to support an astronaut's manual work. The work presented in this paper falls under two individual projects, which have a direct continuum. The first phase was developed in Europeans Space Agency's project called “EdcAR—Augmented Reality for Assembly, Integration, Testing and Verification, and Operations” (Helin, 2017) and the second phase was developed and evaluated within the Horizon 2020 project “WEKIT—Wearable Experience for Knowledge Intensive Training” (Vizzi et al., 2017). The AR system was designed for the Microsoft HoloLens mixed reality platform (Microsoft, 2018) and it was implemented based on a modular architecture. Moreover, the content creation and visualization were designed by the main principles of the IEEE Draft Standard for an Augmented Reality Learning Experience Mode (IEEE: ARLEM, 2018)^1^. The AR system was pre-evaluated in the ISS-Columbus training center located in the ESA's European Astronaut Centre in Cologne, Germany (Tedone et al., 2016). The test team included an experienced astronaut. Updated versions of the final tests were performed on the ISS the Automated Transfer Vehicle (ATV) modules mock-up at ALTEC premises (Vizzi et al., 2017). The current generation of AR can provide immersive experience where human vision and other senses are manipulated in a way that are very believable and seem to alter reality. Wearable Technologies (WT), such as smart glasses, play an important role as delivery devices for such immersive experience (Wild et al., 2017). The objective of this study is to verify if Augmented Reality can be an eligible technology and productive tools in the aerospace industry.

One promising application for AR guidance and training is in maintenance and assembly; also termed AR instructions (Re and Bordegoni, 2014), AR-based job aid (Anastassova et al., 2005), AR-assisted maintenance system and AR-based assembly guidance (Ong et al., 2008). AR guidance means that instructions are given to the user in textual, and/or visual format, augmented on the target objects. The benefits of AR guidance in assembly and maintenance have been noted in several studies: the tasks were easier to handle and they could be done more effectively and with fewer mistakes compared to other instruction formats, and skill transfer could be enhanced (Ong et al., 2008).

User studies in AR contexts have regarded human perception, user task performance, and collaboration between multiple collaborating users, and interface or system usability (Dünser et al., 2008). Users' subjective experiences were most often measured using preference, ease of use, perceived performance, and intuitiveness. The methods in AR user studies have included questionnaires, and/or performance measures (Bai and Blackwell, 2012), although some qualitative measures derived using direct observations, video analysis, and interviews have also been mentioned (Dünser and Billinghurst, 2011). Many user studies have highlighted the potential of AR guidance in work support, however also suggested a number improvements in user experience and design process (e.g., Kuula et al., 2012; Woodward et al., 2014; Helin et al., 2015; Aaltonen et al., 2016). These indicate that user-centric development should be done both in technology and content creation in order to create convincing AR solutions for the end users.

Offering a stimulating and pleasurable user experience (UX) is becoming a central goal and design strategy in the design of digital artifacts and services. The main challenges related to UX can be divided to (1) designing a user experience that is pleasurable, engaging and stimulating, and appropriate in the user's context, and (2) evaluating the UX and overall acceptability of the applications (Olsson, 2013). The ISO standard (ISO 9241–210:2010)^2^ defines UX as “A person's perceptions and responses resulting from the use and/or anticipated use of a product, system or service.” In this study, UX is evaluated using a set of methods including observation, questionnaires and interviews. Furthermore, also aspects of usability are included in the methods. Usability theories focus on pragmatic aspects of product use that furthermore are relatively persistent and at least partly objectively definable for example task completion efficiency, effectiveness and ease-of-use. As for UX, it moves toward a more emotionally appealing relationship between the user and the product (Olsson, 2013). Since the boundary between UX and usability in not always very unambiguous, both pragmatic and emotional aspects are included in the concept of UX in this study.

## AR system for astronaut's manual work support

**Figure 1:**
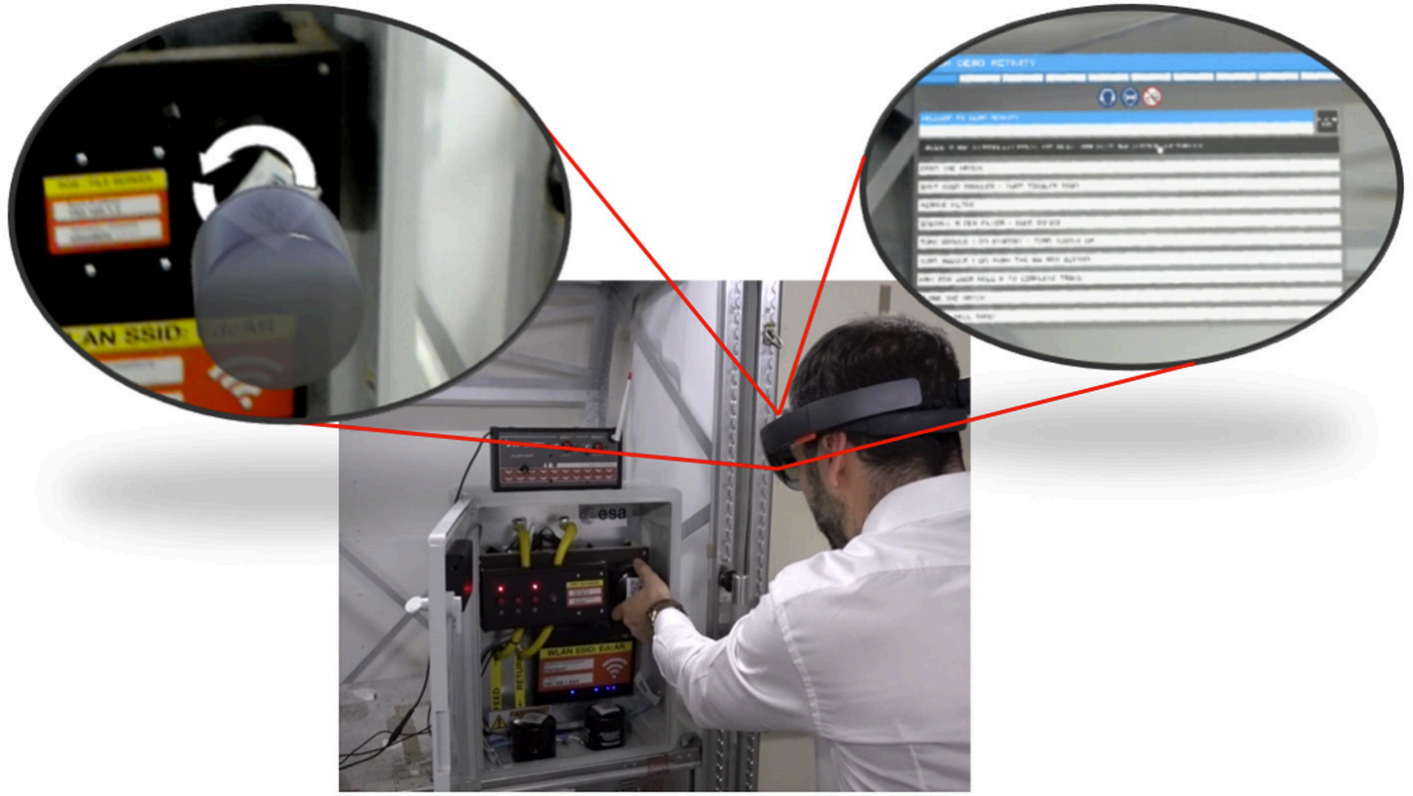
ARLEM based user interface located in 3D space including general warning symbols and 3D models with animation (approved from subject).

**Figure 2:**
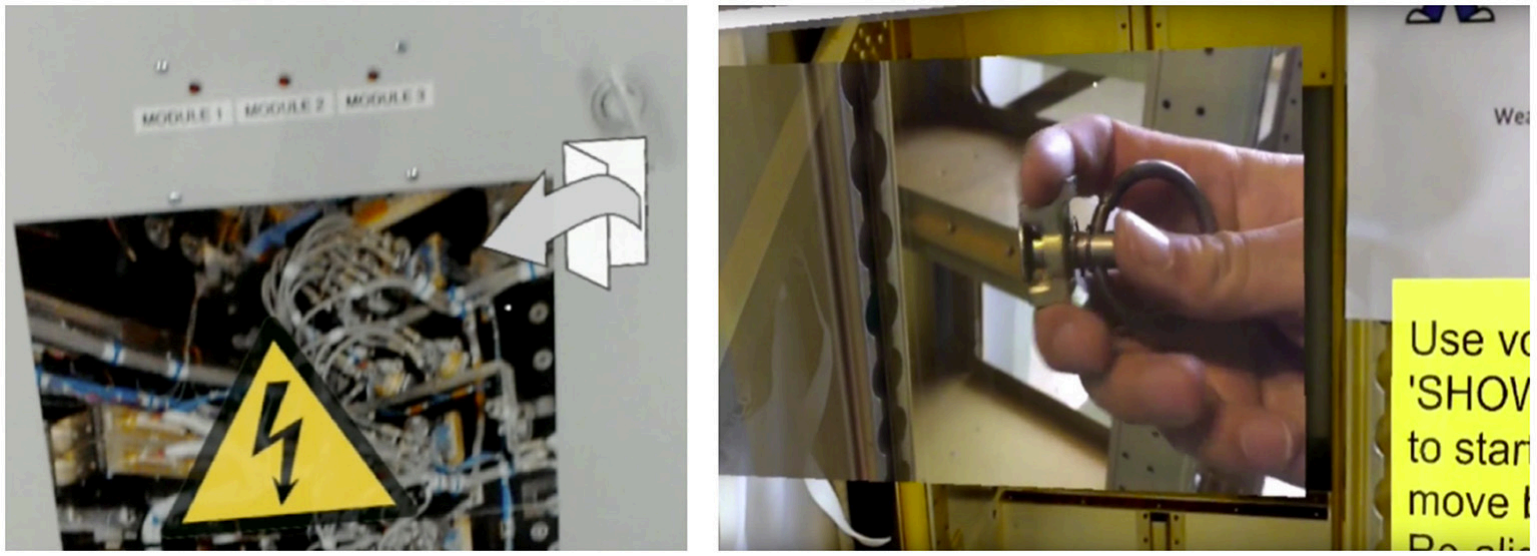
Activity JSON based warning signs and Activity symbols (Left) and video and audio annotation in 3D space (Right).

The AR-system is based on the Microsoft HoloLens mixed reality platform and IEEE Draft Standard for an Augmented Reality Learning Experience Mode. The whole system is configured around the Activity and Workplace JSON files. The Workplace JSON describes workplace-related information such as points of interest, sensors, etc. It is parsed with the Workplace manager and information is transferred to the data layer. The Activity JSON describes all action steps and the content that should be active for each step. It is parsed with the Activity manager and information is transferred to the AR layer via local storage. The current version can annotate:

1. UI in 3D space (Figure 1)
2. Warning symbols (Figure 1)
3. Location based warnings (Figure 2)
4. Symbols (Figure 2)
5. 3D models (Figure 1)
6. 3D animations (Figure 1)
7. Video annotations (Figure 2)
8. Audio annotations (Figure 2)

The user can interact with the AR player via a multi-modal user interface (see Figure 1 top-right corner). The following modalities can be used simultaneously:

- Gesture, e.g., doing a “Click” gesture to go to the next work step.
- Voice commands, e.g., saying “Next” to go to the next work step or “Show status”/“Hide status.”
- Physical HoloLens click button, e.g., “Click” to go to the next work step.
- Physical devices which have an IoT interface e.g., flipping switch to “Stand-by” mode enables IoT constraint in the Activity JSON.

**Figure 3:**
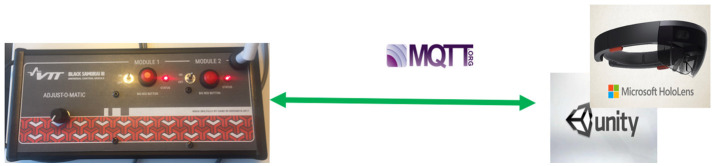
IoT test system with MQTT standard.

**Figure 4:**
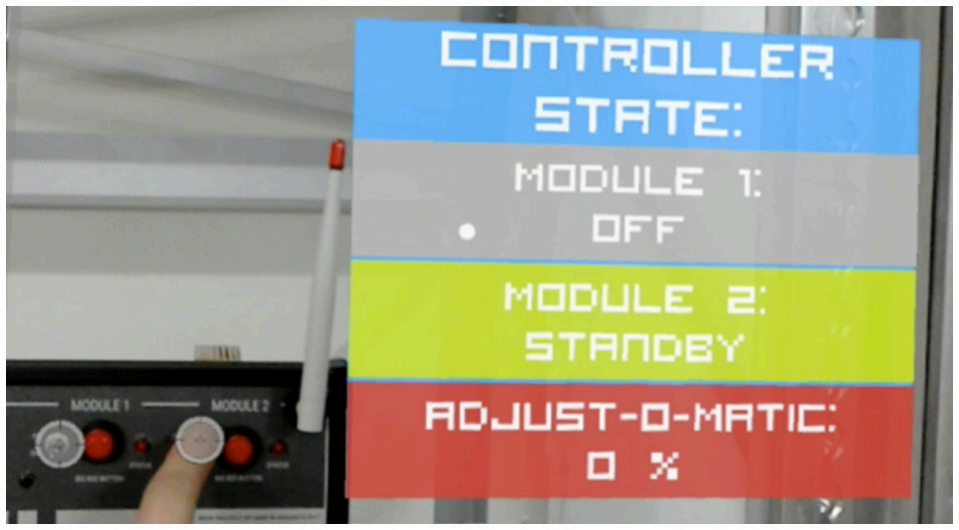
AR-visualization of IoT data with HoloLens.

The main objective of the IoT demo box is to test and demonstrate IoT features in augmented reality. Figure 3 illustrates the main principles of the IoT demo system. The IoT box includes a BeagleBone microcomputer that concurrently runs the MQTT server and the WLAN router. The AR system connects via the MQTT IoT standard and AR-visualizations show information based on IoT data, and Activity and Workplace JSON files (see Figure 4).

## Use cases for system requirements and evaluations

### AR-based centralized cabin filter replacement in ISS

**Figure 5:**
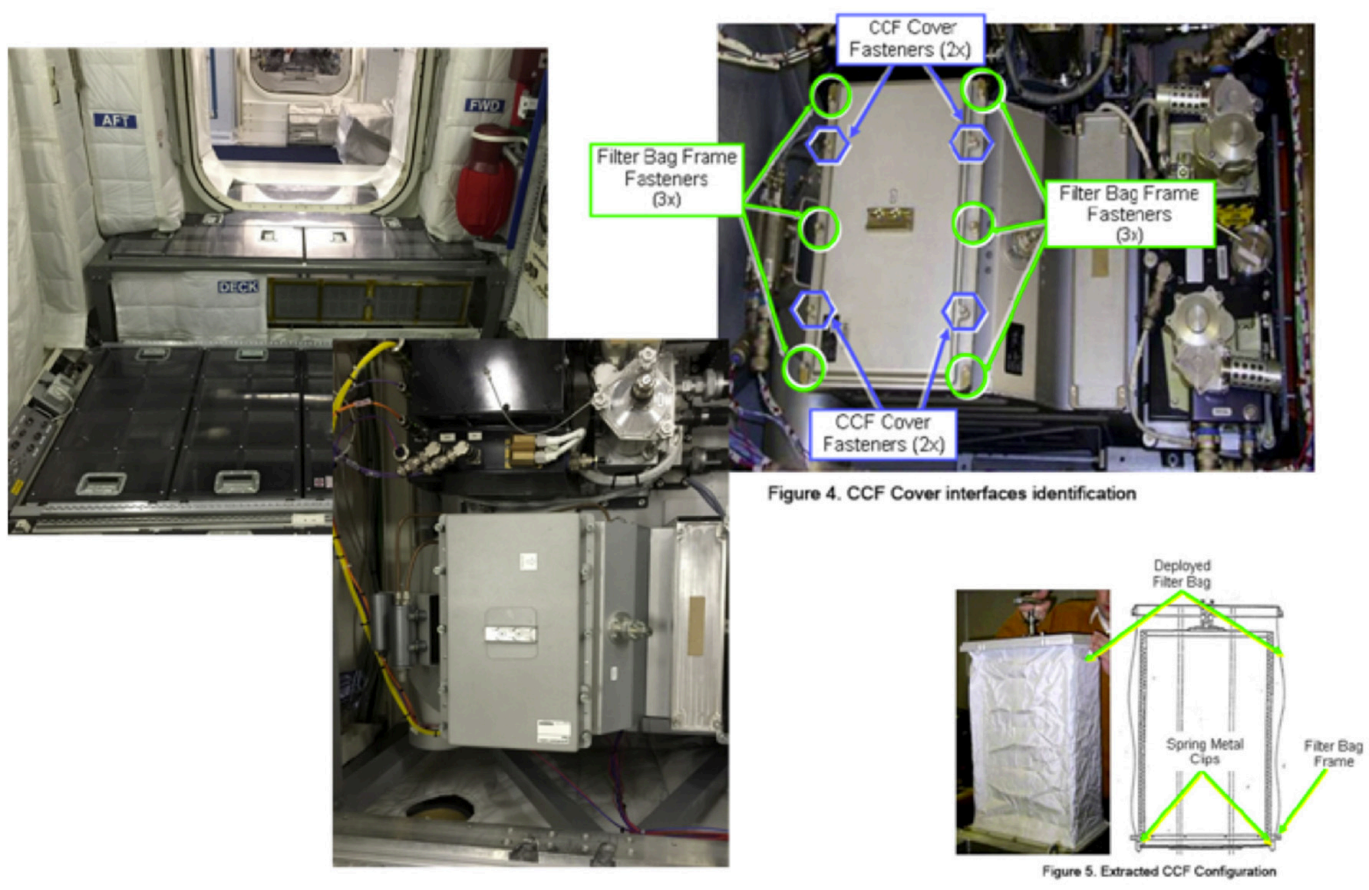
Centralized Cabin Filter Replacement in ISS.

The use case is based on the on-board training and remote support scenario, or more precisely, the Centralized Cabin Filter (CCF) Replacement procedure in International Space Station (see Figure 5). At present, the complete CCF Replacement task takes about 1 h and includes the performance of two referenced procedures (1.201 COL Deck Rack D1/D2/D3 Open & Close and the 2.6.665 Portable O2 Monitor—Sampling Operations). With this use case, we intend to demonstrate that the EdcAR system is able not only to support, but also to instruct the users, on how to perform procedural tasks, in this case the ISS crew (Tedone et al., 2016).

Furthermore, it is understood that the crews of the ISS or other manned space exploration missions will have to face unanticipated situations. At that point, it is important that the crew is able to receive training in real-time in order to perform a required, unforseen task. In this scenario, a possible solution could be that a brand new hands-on lesson, or procedure is developed on the ground with the use of the AR-system, and digitally sent to the ISS, or the spacecraft. The crew, which have never been trained for this specific task will then be able to execute it, thanks to the guidance provided by the AR-system in On-board Training application.

Exploiting the AR-system, the operator will use a tablet that provides a global view of the training scenario and AR glasses for annotated information. For *ad-hoc* remote support, ground control will be able to view video streaming from the astronaut's glasses, as well as tracking information. The following section describes the five-step procedure:

1. The crew wears the AR glasses, takes the physical toolbox and selects a procedure to perform
2. The crew performs the task following instructions given by the AR glasses
3. Columbus Control Centre (COL-CC) supports troubleshooting activities by interacting with the crew over voice and video loops (or watching images of the working area taken by the AR glasses) and by showing directly on the hardware via AR, the parts and the operations to be performed
4. The crew follows the instructions provided by COL-CC through voice and video loops, and the AR system
5. Once the problem is fixed, the crew can continue following the procedure instructions given by AR glasses

### AR-supported installation of automated transfer vehicle in ISS–ATV mock-up

**Figure 6:**
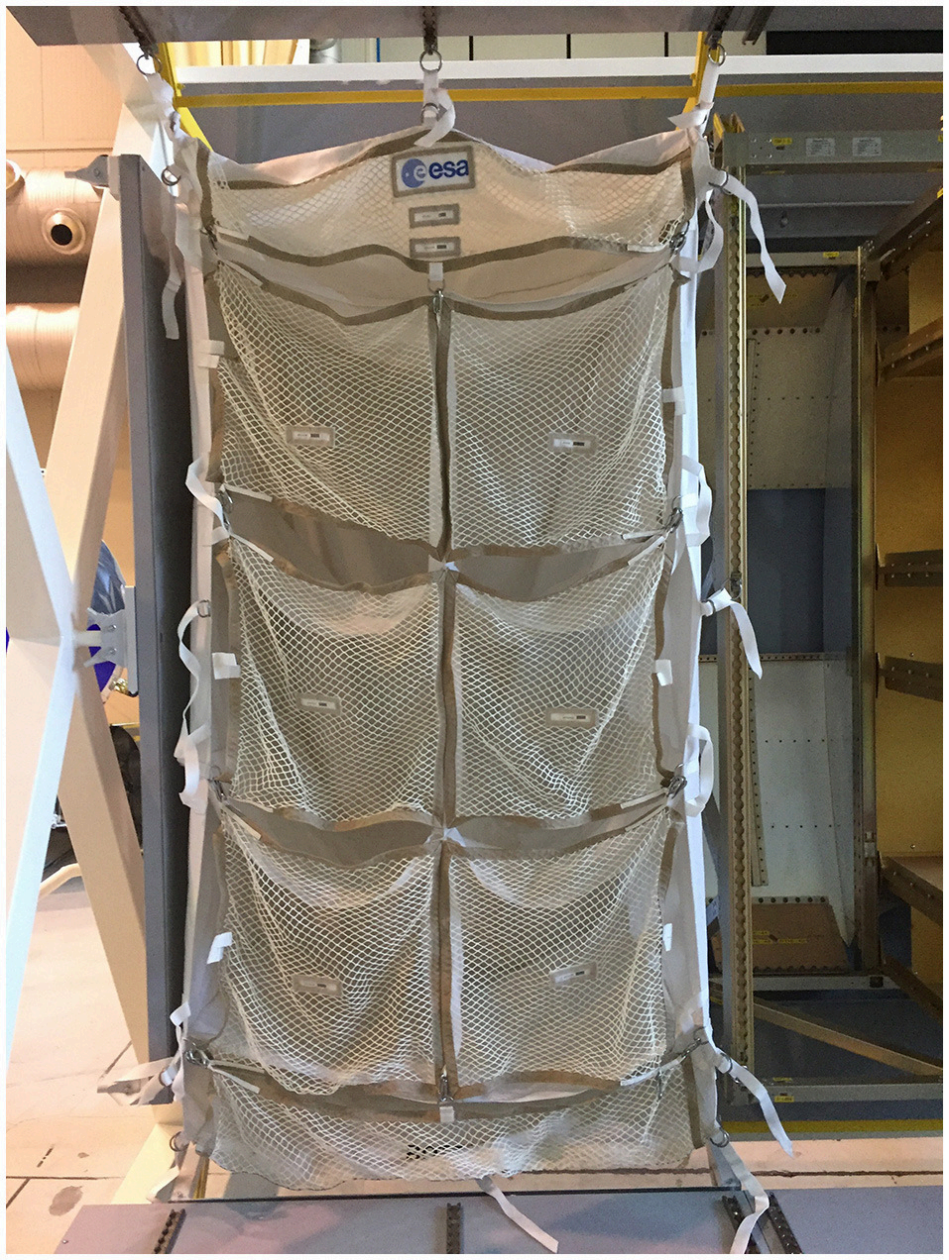
Final result of the TSR after the installation.

The installation of the TSR is a real procedure that the astronauts had to perform on the ISS in the ATV modules. All the components and mock-ups are available at ALTEC facility as well as the complete procedure (see Figure 6). This kind of procedure allows takes into account several aspects of the training methodology, and also to tests the different features of the AR-system prototype.

The AR-system will support the operator while performing the installation of the TSR in the rack. Using the Microsoft HoloLens, the operator will visualize information and data for each step of the procedure by means of videos, audios messages, 3D models, text, and symbols. The steps of the procedure are described below:

1. The operator wears the Microsoft HoloLens and collect the equipment needed to perform the procedure (6 seat track studs with the TSR)
2. The operator executes the procedure following the instructions displayed in the Microsoft HoloLens:
  - Localization of the seat track studs, brackets and ball bearings location through basic augmentation (arrows, circle, etc.)
  - Installation of the studs in the correct position following short video and audio that explains how to use them
  - Orientation of the TSR in the correct position supported by images
  - Connection of the straps to the interfaces following short video and audio that explains how to fix the straps
3. Once the installation is complete, the operator tightens the straps to fix the TSR to the rack
4. The operator check that the installation has been completed successfully comparing the final result with a 3D model that is over imposed on the working area.

## Evaluations

### Performed evaluation cases

**Figure 7:**
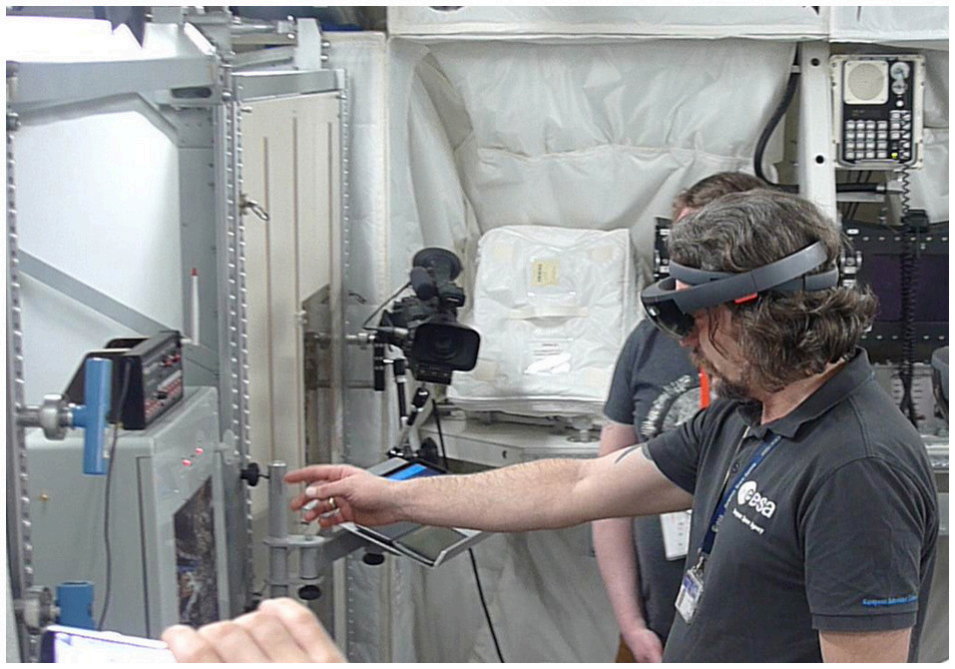
EAC personnel are testing the updated version (approved from subject).

The first evaluation (EdcAR) of the AR-system has been performed in the ISS-Columbus training mock-up located in the ESA's European Astronaut Centre in Cologne, Germany. The test group included 14 subjects, an experienced astronaut, EAC trainers, other EAC personnel, and a student group. During the test of the AR-system all test subjects were able to test the Microsoft HoloLens based system. Figure 7 shows the testing scenario, EAC personnel is testing the AR-system.

**Figure 8:**
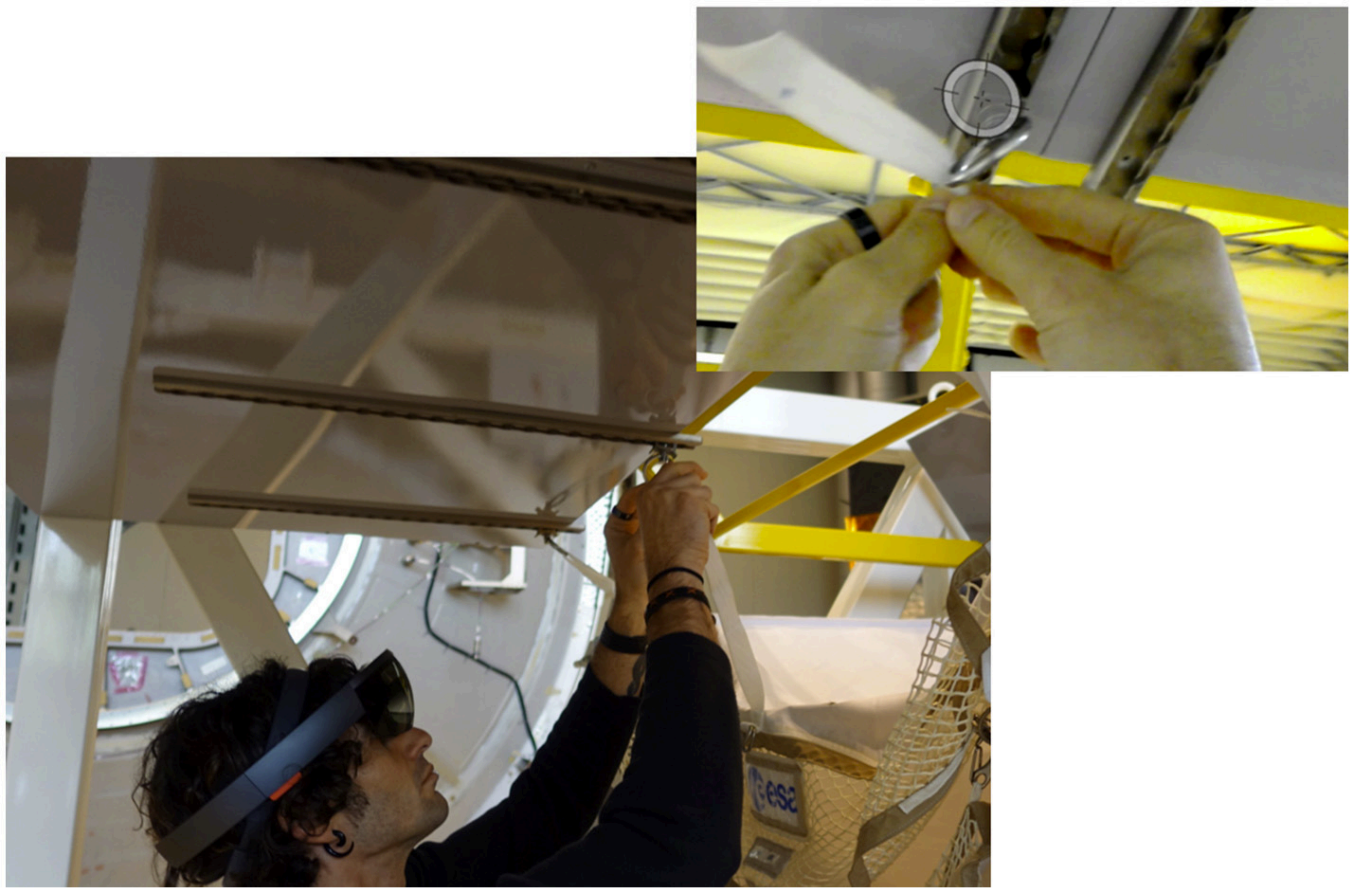
The TSR installation at ALTEC premises (approved from subject).

The second evaluation of AR-system was tested at ALTEC facility (Turin, Italy) performing a real astronaut procedure on a physical mock-up of an ISS module (see Figure 8). Thirty-nine participants took part in the test: 17 experts from ALTEC and Thales Alenia Space Italy and 22 students from the International II level Specializing Master in Space Exploration and Development Systems (SEEDS) and Politecnico di Torino.

### Evaluation methods

#### Observation and expert evaluation

In the first project (EdcAR), the development team evaluated the user experience and usability by observing the user test. The observations were written down while the users were testing the system, based on the comments, and performances of the users. The idea of the evaluation was to allow the users comment freely their experience, without predetermined questions or framework, and thus let the users define what is significant for them. The approach was applicable also because of the small amount of test users. After the test, the development team summarized the written observations and drew the conclusions.

#### Questionnaires

For the second test (WEKIT trial), a set of questionnaires was used to collect feedback from the participants and to enable the evaluation process after the trial. Of the original set of seven separate questionnaires, the results from three UX-related are presented in this paper. The questionnaires include System Usability Scale (SUS), Smart Glasses User Satisfaction (SGUS) and User Interaction Satisfaction (QUIS).

#### System usability scale

SUS is a tool for measuring both usability and learnability. The SUS scores calculated from individual questionnaires represent the system usability. SUS yields a single number representing a composite measure of the overall usability of the system being studied. Scores for individual items are not meaningful on their own. SUS scores have a range of 0 to 100 (Brooke, 1996, 2013). According to validation studies, the acceptable SUS score is about 70 (Bangor et al., 2009; Brooke, 2013).

#### Smart glasses user satisfaction

SGUS questionnaire was created for the WEKIT trials. SGUS measures subjective satisfaction focusing especially on test participants' experiences on the features that support learning. SGUS is based on the evaluation criteria for web-based learning by Ssemugabi and de Villiers (2007) and statements taken from Olsson (2013) “Concepts and Subjective Measures for Evaluating User Experience of Mobile Augmented Reality Services.” SGUS consists of 11 items (statements) with a seven point Likert scale (1–7).

#### The questionnaire for user interaction satisfaction

QUIS measures subjective satisfaction with specific aspects of the interface, including usability and user experience (Chin et al., 1988). QUIS was modified, using only the relevant items from the viewpoint of this study. Altogether 15 QUIS items with a scale mapped to numeric values of 1 to 7 were used.

#### Interviews

Additionally to the questionnaire, six (6) participants were interviewed during the space case trial. Two (2) of the interviewees were experts and four (4) were students. The participants to be interviewed were chosen randomly according to the availability of the participants and to avoid wasting their time (i.e., in a group of 3 people, the first one who finished was interviewed while waiting for his/her colleagues to finish the test). The interviews consisted of four main questions or themes: (1) Please use 2–3 words (adjectives) to describe your experience with AR glasses and the player during the execution of the task; (2) Please describe freely your experience on using the player during the execution of the task; (3) How well did you manage to complete the task with the player?; (4) Please tell the good and bad sides of AR glasses (HoloLens). Two to three positive and negative aspects.

### Ethics approval

This study was carried out in accordance with the recommendations of Norwegian Centre for Research Data and VTT's Code of Conduct, which includes Ethical guidelines. All subjects gave written informed consent in accordance with the Declaration of Helsinki.

## Results

### Observation and expert evaluation results

Based on the observation and expert evaluation of the ISS-Columbus mockups use-case, the HoloLens based AR-system is usable for basic daily use as its usability has reached a reasonable level. The 3D space user interface and annotations were working properly without any delay and the image quality was high enough for see-through glasses. The main downside of the system is the narrow field of view, the user has to move his/her head around and search for the information in some cases. Test subjects also highlight the potential of applying AR technology to this field, but AR-systems UI should be update to support Operations Data File (ODF) format which is common in the space domain.

### Questionnaire results

**The System Usability Scale (SUS)** questionnaire showed that the SUS scores were very close to 70 (scale 0–100), which is considered as the minimum acceptable score for system usability. Thus, the WEKIT applications were considered nearly acceptable in terms of system usability. There is a clear difference between student and expert groups in deviation, indicating that there was a greater variation in the experience of system usability in student group. The lowest SUS score in a student group was 23 and the highest was 95 (see Table 1).

**Table 1:** SUS results for the Space trial.

**Group**	**SUS score average**	**Standard deviation**
Experts	69,2	10,6
Students	67,5	18,3
All	68,3	15,1

Based on **Smart Glasses User Satisfaction (SGUS)** questionnaire results, the participants experienced that the system and content helped them to accomplish the task quite well (GL7) and their attention was captivated in a positive way (GL6). The provided content was also contextually meaningful (GL2). However, using the AR glasses was experienced as less natural (GL4, GL9), and following and understanding the task phases (GL8, GL10) not very easy. The overall average (5,4) was satisfactory on a scale 1–7 (see Table 2).

**Table 2:** SGUS results for the Space trial.

**SGUS ITEM**	**Mean**	**St.dev**	**Items above overall average 5,4**
GL1 With AR-glasses I could access information at the most appropriate place and moment.	5.1	1.2	
GL2 Content displayed on the AR-glasses made sense in the context I used it.	5.7	1.0	X
GL3 AR-glasses provided me with the most suitable amount of information.	5.3	1.1	
GL4 AR-glasses allowed a natural way to interact with information displayed.	5.0	1.5	
GL5 I had a good conception of what is real and what is augmented when using AR-glasses.	6.0	1.1	X
GL6 The interaction with content on AR-glasses captivated my attention in a positive way.	6.0	0.8	X
GL7 The instructions given by AR-glasses helped me to accomplish the task.	5.9	1.0	X
GL8 I understood what is expected from me in each phase of the task with the help of AR-glasses.	4.9	1.4	
GL9 Performing the task with the help of AR-glasses was natural to me.	4.8	1.6	
GL10 While using AR-glasses, I was aware of the phase of the task at all times during the execution of the task.	5.2	1.5	
GL11 While using AR-glasses, I was able to pay attention to the essential aspects of the task all the time.	5.6	1.1	X

The analysis of the Questionnaire for **User Interaction Satisfaction (QUIS)** showed that the scores were close to the average score for most of the questions. Taking a closer look at scores above or below the average, it was possible to assess that the current (HoloLens) version of AR is not yet considered fully reliable by the users, where the characters on display were found a bit hard to read and the organization of the information on the display was a bit too confusing and inconsistent. These results were observed in particular in the answers of the students.

On the other hand, the experience with AR glasses for performing that kind of task was considered good for both the experts, and the students, that found it easier to learn how to operate on such a task being guided by AR instructions (see Table 3).

**Table 3:** QUIS results for the Space trial.

**QUIS item**	**Mean all**	**St.deviation**	**Items above overall average 5,1**
QS1 Terrible - Wonderful	5,7	1,2	X
QS2 Difficult - Easy	5,2	1,5	X
QS3 Frustrating - Satisfying	5,1	1,3	
QS4 Inadequate power - Adequate power	5,2	1,1	X
QS5 Dull - Stimulating	6,1	0,8	X
QS6 Rigid - Flexible	4,4	1,2	
QS7 Unreliable - Reliable	5,0	1,4	
QS8 Slow - Fast	5,1	1,1	
QS9 Characters on the display Hard to read - Easy to read	4,6	1,6	
QS10 Organization of information on the display: Confusing - Very clear	4,8	1,4	
QS11 Positioning of messages: Inconsistent - Consistent	4,4	1,3	
QS12 Messages on screen which prompt user for input: Confusing - Very clear	5,0	1,2	
QS13 Learning to operate the glasses: Difficult - Easy	5,6	1,6	X
QS14 Exploring new features by trial and error: Difficult - Easy	5,3	1,3	X
QS15 Tasks can be performed in a straightforward manner: Never - Always	5,1	1,0	

### Interview results

As the results of the interviews (*N* = 6), participants' overall experience on the system, the experienced pros and cons of the Microsoft HoloLens and improvement suggestions for UX are presented in this section.

#### Participants' overall experience on the system

Participants' positive and negative descriptions are listed in Table 4, based on interview question “Please use 2–3 words (adjectives) to describe your experience with AR glasses and the player during the execution of the task.”

**Table 4:** Participants' overall experience.

**Positive descriptions**	**Negative descriptions**
Interesting (4 times)	Frustrating (2 times)
Useful (2 times)	Embarrassing
Easy (2 times)	
Fun	
Nice	
Engaging	
Quite innovative	
Great	
Addictive	
Stimulating	

The overall experience on the AR system was quite positive for the interviewees, since only three negative descriptions resulted from the interviews. The experienced frustration was related to the lagging of the system and speech recognition. The commands had to be repeated several times, probably because of improper pronunciation. Because of repetition of commands, the task sequence sometimes moved on two steps instead of one. Additionally, it was difficult to know where to look especially when looking for video guidance. Watching or finding the visual cues caused some problems. For one interviewee, this caused problems during the task performance, which the person defined as embarrassing. Audio instructions seemed to create mixed opinions: some participants appreciated them while others found visuals are better than audio.

#### The pros and cons of the microsoft hololens and improvement suggestions for UX

The experienced pros and cons of the HoloLens are presented in Table 5, based on the question “Please tell the good and bad sides of AR glasses (HoloLens). Two to three positive and negative aspects.”

**Table 5:** The pros and cons of the Microsoft HoloLens.

**Pros**	**Cons**
Comfortable (2 times)	Vision is limited / narrow view (3 times)
Engaging (2 times)	Bulky
Light	Doesn't look that cool
Useful	Too complicated
Interactive	It didn't understand me sometimes (speech recognition)
Futuristic	The tasklist was too far
Easy to use	Text fonts hard to read, size is ok
Worked better than I thought, the way it followed my movements	Placement of content is obstructive
Tracking is really good	Have to be careful of real world collisions and objects in case of accident. Needs warnings when moving. Needs clear space without hazards.
Gesture recognition	Negative I don't have
Resolution	For creating recording, the process could be more intuitive
Overall task was easy to perform	
Useful to do without Instructor	
Recording gives several tools that are useful for building a training session	
Overlap of Virtual and Real (AR) is good	
Involving	

The field-of-view was experienced as too limited and narrow during the task execution. It seemed to be difficult to perceive the whole task area and also to understand where to look for instructions and information on the next steps in the tasks sequence.

The interviewees' suggested improvements for user experience are listed below:

- Help function could be useful
- Better cues for where to look for information and instructions. It was also suggested that the video should be adjusted with the head position and movement
- Possibility to go back / return in the task steps sequence
- The system should give feedback on the task performance
- Sometimes the visual cues were too close to the actual object. Probably a smaller view of the task area could be an improvement
- The speech commands should be considered critically and possibly provide other possibilities
- The font type and size should be considered more carefully

## Conclusions and discussion

The evaluation results of user experience in two AR user studies are presented in this paper. The main emphasis was on data collection and analysis of the EU WEKIT project case with real astronaut installation procedure on a physical mock-up of an ISS module.

The aim of both studies was to develop and improve an AR system for astronauts' manual work and these studies are closely linked. The number of test participants in the first case (EdcAR) was 14 and in the second case (WEKIT) 39. According to the recent review of AR usability studies (Dey et al., 2018), 12 to 18 participants per study is a typical range in the AR community. While the first user study falls into this category, the second differs significantly with a higher number of participants. However, the two studies can be considered as phases of one larger study, and thus with the complete number of over 50 participants, this study provided a good ground for evaluating the user experience on this stage of prototype development.

Based on the results, the overall user experience of the AR system is quite satisfactory. The most common descriptions of the whole system or the HoloLens are “interesting,” “useful,” “easy,” “engaging” and “comfortable,” whereas the questionnaire results indicate that the system has a very strong potential; to e.g., captivate attention positively and support task accomplishment effectively.

As the AR-system usability has reached a reasonable level (average SUS score 68), both the pragmatic and emotional aspects of the user experience were considered fulfilling. It can be suggested that the AR-system is potentially a useful tool for supporting and facilitating the assembly and training procedure in the space field, even though the tool is still in prototyping phase and it will be updated after the next trials in WEKIT project.

There are however many usability issues still to be resolved. For instance, the narrow field-of-view is one the most significant issues.

On the other hand, the experienced problems in perceiving the work area and finding or noticing AR objects could be solved by enhancing visual guidance (e.g., 360° guidance and animated lines for locating objects), providing clear orientation to the task and by providing instructive materials of the area (maps, 3D models etc.)

From the technical point-of-view, the main indications to operate the AR-system inside the ISS-Columbus module on orbit were investigated and aspects related to the system integration were considered: (1) in order to operate the proper selection of the COTS items requesting only minor changes for operations in the ISS, (2) in order to highlight possible criticisms and critical technologies to be further investigated in the prototype design, assembly and testing phases, and (3) in order to address the design of the flight concept.

The developed system has been already tested and evaluated in two other domains: bioimaging and aircraft maintenance. These tests were completed together with the EU-WEKIT project. Microsoft HoloLens AR-system worked properly and its usability has a reasonable level for performing the required task. Over 40 persons from another domain evaluated the AR-system. This shows that current system is also suitable for other domains.

## Ethics statement

This research has been following VTT's Code of Conduct, which includes Ethical guidelines. VTT's ethical committee: Matti Karhunen (chair), Seppo Viinikainen, Reetta Grenman and Richard Fagerström.

## Author contributions

KH: AR system description, EdcAR case and evaluation, conclusion; TK: Evaluation methods and results, conclusion; CV: ALTEC case description; JK: AR system description; AV: Evaluation methods and results.

### Conflict of interest statement

The authors declare that the research was conducted in the absence of any commercial or financial relationships that could be construed as a potential conflict of interest. The reviewer JV and handling Editor declared their shared affiliation.

## Footnotes

^1^*P1589-IEEE Draft Standard for an Augmented Reality Learning Experience Mode*. Available onlile at: https://standards.ieee.org/develop/project/1589.html (Accessed April 21, 2018)

^2^Ergonomics of human-system interaction-Part 210: Human-centred design for interactive systems.
